# Evaluation of conventional and four real-time PCR methods for the detection of *Leishmania* on field-collected samples in Ethiopia

**DOI:** 10.1371/journal.pntd.0008903

**Published:** 2021-01-12

**Authors:** Behailu Merdekios, Myrthe Pareyn, Dagimawie Tadesse, Nigatu Eligo, Mekibib Kassa, Bart K. M. Jacobs, Herwig Leirs, Jean-Pierre Van Geertruyden, Johan van Griensven, Guy Caljon, Lieselotte Cnops

**Affiliations:** 1 Department of Public Health, Arba Minch University, Arba Minch, Ethiopia; 2 Evolutionary Ecology Group, University of Antwerp, Antwerp, Belgium; 3 Department of Medical Entomology, Arba Minch University, Arba Minch, Ethiopia; 4 Leishmaniasis Research and Treatment Centre, University of Gondar, Gondar, Ethiopia; 5 Department of Clinical Sciences, Institute of Tropical Medicine, Antwerp, Belgium; 6 Global Health Institute, University of Antwerp, Antwerp, Belgium; 7 Laboratory of Microbiology, Parasitology and Hygiene, University of Antwerp, Antwerp, Belgium; Charité University Medicine Berlin, GERMANY

## Abstract

In most low-resource settings, microscopy still is the standard method for diagnosis of cutaneous leishmaniasis, despite its limited sensitivity. In Ethiopia, the more sensitive molecular methods are not yet routinely used. This study compared five PCR methods with microscopy on two sample types collected from patients with a suspected lesion to advise on optimal diagnosis of *Leishmania aethiopica*. Between May and July 2018, skin scrapings (SS) and blood exudate from the lesion spotted on filter paper (dry blood spot, DBS) were collected for PCR from 111 patients of four zones in Southern Ethiopia. DNA and RNA were simultaneously extracted from both sample types. DNA was evaluated by a conventional PCR targeting ITS-1 and three probe-based real-time PCRs: one targeting the SSU 18S rRNA and two targeting the kDNA minicircle sequence (the ‘Mary kDNA PCR’ and a newly designed ‘LC kDNA PCR’ for improved *L*. *aethiopica* detection). RNAs were tested with a SYBR Green-based RT-PCR targeting spliced leader (SL) RNA. Giemsa-stained SS smears were examined by microscopy. Of the 111 SS, 100 were positive with at least two methods. Sensitivity of microscopy, ITS PCR, SSU PCR, Mary kDNA PCR, LC kDNA PCR and SL RNA PCR were respectively 52%, 22%, 64%, 99%, 100% and 94%. Microscopy-based parasite load correlated well with real-time PCR Ct-values. Despite suboptimal sample storage for RNA detection, the SL RNA PCR resulted in congruent results with low Ct-values. DBS collected from the same lesion showed lower PCR positivity rates compared to SS. The kDNA PCRs showed excellent performance for diagnosis of *L*. *aethiopica* on SS. Lower-cost SL RNA detection can be a complementary high-throughput tool. DBS can be used for PCR in case microscopy is negative, the SS sample can be sent to the referral health facility where kDNA PCR method is available.

## Author summary

Cutaneous leishmaniasis is a neglected tropical disease and causing a public health problem in Ethiopia. Microscopy is still the standard method for detection of the parasite in Ethiopia, and also in many other low resource settings. A more sensitive method is needed for timely diagnosis and treatment. In this study, we compared five molecular methods on samples collected from patients with a skin lesion suspected of cutaneous leishmaniasis to advice on optimal diagnosis of *L*. *aethiopica*. We collected two sample types from the same lesion (skin scrapings and lesion fluid on filter paper) and isolated both DNA and RNA of them. Majority (90.1%) of the samples from skin scrapings were positive in two or more methods and the molecular methods had a higher sensitivity than the conventional methods. Interestingly, we evaluated for the first time a new molecular method designed to improve *L*. *aethiopica* detection. Also, we showed that RNA detection performed well for samples that were collected under difficult field conditions. Samples collected on filter paper showed less positive results than skin scraped samples, but could still be the method of choice for easy sampling and transport in resource-limited settings as it performed better than microscopy.

## Introduction

Cutaneous leishmaniasis (CL) is a vector-borne disease caused by parasites of the genus *Leishmania*, which are transmitted by the bite of infected female phlebotomine sandflies. CL is endemic in more than 80 countries globally with an estimated 0.7–1.2 million CL cases each year, predominantly in 4 countries of the New World and 6 of the Old World (including Ethiopia) together accounting for 70 to 75% of global CL incidence [1]. More than 20 different *Leishmania* species can cause CL with some that geographically coexist. *Leishmania (L*.*) major* and *L*. *tropica* are most common in the Old World. In Ethiopia, there is a unique dominant species, *L*. *aethiopica*, which is mainly found in the highlands putting nearly 29 million populations at risk and has an annual burden of an estimated 20,000 to 50,000 cases per year [1–4].

CL is characterized by slowly growing nodular or ulcerative lesions, typically healing with scars. While not life-threatening, lesions can be disfiguring and stigmatizing, particularly those occurring in the face [5]. Localized CL is the most common clinical form, predominantly affecting the face, but also mucocutaneous CL and to a lesser extent diffuse CL are regularly reported in Ethiopia. In contrast to New World CL, *L*. *aethiopica* typically causes crusty lesions with a patchy distribution and local edema that slowly develop and heal eventually (requiring approximately one to three years). However, sometimes the infection may progress to more severe, chronic and complicated forms [6–8].

Like in many other resource-constrained countries, microscopic examination of Giemsa stained skin scrapings (SS) is still the cornerstone for CL diagnosis in Ethiopia. However, it is increasingly recognized that its sensitivity is suboptimal, ranging from around 17–83% [6, 9, 10], and is heavily dependent on technical expertise, staining quality, lesion type, and reference test used to determine the sensitivity. Molecular methods, such as polymerase chain reaction (PCR), combine high sensitivity with high specificity. Even in resource-constrained settings, user-friendly PCR platforms are nowadays well-established for routine diagnosis and surveillance of tuberculosis [11] and HIV [12]. Although such molecular tools have the potential to be used for other neglected diseases like leishmaniasis as well [13, 14], there is still a long way to go before implementation in routine care. Ideally, PCR testing should facilitate both diagnosis and surveillance. For the latter, easier tools for sample collection and/or storage would be useful. For example, filter paper is increasingly used for sample collection in remote settings with subsequent centralized analysis at a later stage [15–18].

PCRs designed in either conventional or real time formats can target different regions of the *Leishmania* genome for parasite detection at the genus, complex or species level [19, 20]. Typically, these PCRs target nuclear and ribosomal DNA, like the small subunit (SSU) 18S ribosomal RNA or internal transcribed spacer (ITS) ribosomal regions [21, 22] or the mini-exon spliced leader (SL) gene repeat [23, 24]. Another commonly used target is the extra-chromosomal minicircle kinetoplast DNA (kDNA), which is present in several thousands of copies, resulting in a considerably higher sensitivity [25, 26]. Alternatively, the parasite RNA can be detected which is considered as a marker for viable parasites, such as with the recently developed RT-PCR targeting the SL RNA sequence. This molecular target is conserved in *Leishmania* and performed well on *L*. *infantum* infected hamsters, spiked human blood and clinical samples from visceral leishmaniasis patients [27], but is not yet evaluated on CL patients.

With several targets and diagnostic methods available, it can be difficult to select the PCR method that is optimal for a particular setting. In the context of CL in Ethiopia, the PCR method must be applicable on different clinical sample types, capable of detecting *L*. *aethiopica* and more sensitive than microscopy. The aim of this study was to compare microscopy with five different molecular methods on two different sample types collected from skin lesions of suspected CL patients in the south of Ethiopia and to discuss their potential as diagnostic and surveillance tool in endemic settings.

## Methods

### Ethics statement

This survey was ethically approved by the Institutional Ethical Review Committee of the College of Medicine and Health Sciences of Arba Minch University, Ethiopia (Letter Ref No: CMHS/1167/111 dated 18^th^ April 2018). Samples were collected from all volunteers who gave their oral consent to participate in the study.

### Sample collection

An active case finding survey was conducted between May and July 2018, in which participants suspected of CL were conveniently selected from the 38 rural kebeles in four zones (Gamo Goffa, 27; Wolayta, 5; Dawuro, 3 and Segen area, 3) of the Southern Nations Nationalities and Peoples’ Regional State of Ethiopia. Samples were collected from 111 suspected CL patients who gave their oral consent to participate in the survey. For collection of the samples, one small incision was made with the point of a surgical blade at the margin of the lesion after it was cleaned with 70% denatured alcohol. Two types of samples were subsequently collected: (i) a skin scraping (SS) collected along the cut edge of the incision of which one part was stored in 97% ethanol at -20°C for PCR analysis and the second part was smeared onto two glass slides for microscopy, and (ii) a blood exudate from the same lesion spotted on filter paper, further referred to as dry blood spot (DBS). The blood exudate for DBS collection was immediately taken from the SS incision with a capillary and dropped onto two Serobuvard calibrated pre-punched filter paper disks (LDA, Zoopole, Ploufragan, France) until saturation (approximately 5 μl/disk).

### Microscopy

After dried completely, the skin smears were fixed with 100% methanol, dried again and stained with 5% Giemsa for microscopic examination [28]. The slides were observed under a light microscope with a 1000*×* magnification. The examination of duplicate smears was carried out blindly by two experienced staff members, and thereafter, results were compared to each other. In case of a discordant result between the two readers, a third expert observed the slides and a consensus result was reached by a two out of three observers’ agreement. Parasite load was graded from +1 to +6 according to WHO parasite grading standard operating procedure [29].

### DNA/RNA extraction

Before the extraction procedure, the SS were centrifuged, the ethanol was removed and the remaining tissue was left to dry. DNA and RNA were simultaneously extracted from the SS pellet and DBS using the NucleoSpin RNA kit and an additional NucleoSpin RNA/DNA buffer set (Macherey Nagel, Germany). This protocol enabled sequential elution of DNA and RNA from a single sample. The isolation from DBS was done slightly different than mentioned in the standard manufacturer’s protocol: one pre-cut circle was incubated in β-mercaptoethanol (for RNase inactivation) and lysis buffer for 3 hours at room temperature with frequent vortexing to elute the blood from the filter paper. Eventually, RNA was eluted in 60 μl nuclease-free water while DNA was eluted in 100 μl DNA elute. Both extracts were stored at -20°C until further analysis in the laboratories of Arba Minch University by conventional PCR and Gondar University by real-time PCR.

### Conventional ITS-1 PCR

DNA isolates of the SS samples were subjected to a conventional PCR targeting a 350 bp fragment of the ITS-1 gene (“ITS PCR”), based on El Tai *et al*. [22] as described before [30]. In short, the samples were screened in duplicate with a 15 μl reaction mix consisting of 0.5 μM of each primer (LITSR 5’-CTGGATCATTTTCCGATG-3’ and L5.8S 5’-TGATACCACTTATCGCACTT-3’ (Invitrogen, Life Technologies, Belgium)), 0.2 mM dNTP (GE Healthcare Lifescience, Belgium), 1X QIAGEN PCR Buffer (Qiagen, Belgium), 0.04 U/μl HotStarTaq DNA polymerase (Qiagen) and 1.5 μl of 1/10 diluted DNA extract. The reaction was carried out on a Biometra T professional gradient Thermocycler (Biometra, the Netherlands) and amplicons were visualized on a 1.5% agarose gel. A negative (no template) and positive (*L*. *aethiopica* infected *Phlebotomus pedifer* DNA extract) control were used for each run.

The ITS-1 amplicons were also used to identify the *Leishmania* species in a selection of positive samples from six areas: five from Gamo Goffa (Zadha, Kemba, Demba Goffa, and Kucha woredas) and one from Wolayta (Kindo-Didaye woreda). Amplicons were sent to Vlaams Instituut voor Biotechnologie (VIB) at the University of Antwerp (Belgium) for Sanger sequencing. The obtained sequences were aligned in GenBank using the BLAST tool and the *Leishmania* species was identified if query coverage and identity exceeded 98%.

### Real-time PCR assays

DNA extracts were also tested with three TaqMan probe-based real-time PCRs: one targeting the SSU 18S rRNA gene (referred to as ‘SSU PCR’) and two targeting the kDNA minicircle sequences (the first here so-called ‘Mary kDNA PCR’ that was originally designed by Mary *et al*. for *L*. *donovani* complex species for VL [26]; and the second further called ‘LC kDNA PCR’ that was newly designed to improve *L*. *aethiopica* detection).

The SSU PCR used primers (18S-L-F and 18S-L-R; 0.4 μM) as described by Deborggraeve *et al*. [31] with an additional 18S probe (0.1 μM) as described before [30] and the Mary kDNA PCR was performed with the primers (0.6 μM of each primer) and hydrolysis probe (0.4 μM) as described [26]. The LC kDNA PCR makes use of primers from Nuzum *et al*., [13] that were adapted to forward primer LC-F (5’-TATTTTACACCAACCCCCAGT-3’; 1 μM) and reverse primer LC-R (5’-GGTAGGGGCGTTCTGC-3’; 1μM) with a newly designed FAM-labeled LC-probe (5’-CAGAAAYCCCGTTCAAAAAATGGC-3’, 0.4 μM).

Technical validation of the LC kDNA PCR was performed by testing reactivity with all Old World *Leishmania* species (*L*. *aethiopica*, *L*. *tropica*, *L*. *major*, *L*. *infantum*, *L*. *donovani*) and New World reference strains (*L*. *braziliensis*, *L*. *mexicana*, *L*. *amazonensis*, *L*. *peruviana*, *L*. *panamensis*, *L*. *guyanensis*, *L*. *lainsoni*). The analytical sensitivity was determined based on serial dilutions of four *L*. *aethiopica* strains. Cross-reactivity was also assessed for *Trypanosoma brucei gambiense*, *Trypanosoma brucei rhodesiense*, *Mycobacterium leprae*, *Mycobacterium lepromatosis* and *Plasmodium falciparum*. In addition, analytical specificity was tested on whole blood samples of 25 endemic controls (from Ethiopia) and 10 healthy non-endemic controls (from Belgium).

The three PCRs were run with HotStarTaq Master mix kit (Qiagen) in a total volume of 25 μL containing 1x master mix, primers and probes (Integrated DNA Technologies (IDT), Leuven, Belgium), 4.5 mM MgCl_2_ (SSU PCR only), 0.01% BSA (Roche, Vilvoorde, Belgium), and 5 μL DNA. The PCR programme consisted of an initial activation step of 15 min at 95°C, followed by 50 cycles of denaturation for 5 sec at 95°C, annealing for 20 sec at 58°C, and elongation for 30 sec at 72°C on the RotorGeneQ cycler (Qiagen). A fixed and stringent fluorescent threshold (0.2) was used to determine the cycle threshold (Ct) value.

### Real-time RT-PCR assay

RNA extracts were diluted 1/10 and subjected to the SYBR green-based reverse transcriptase (RT)-PCR targeting the spliced leader RNA sequence (SL RNA PCR) as described [27]. The SL RNA PCR was also run on the RotorGeneQ cycler. In the absence of a melt curve analysis, a stringent Ct-value cut-off of 32.9 was applied for the positive identification of SL RNA amplicons. This cut-off was established based on a ROC analysis on historical RT-PCR data (82 negatives and 81 VL-positive samples), providing 98% sensitivity and specificity.

In each real-time PCR run, two no-template negative PCR controls (PCR-grade water and elution buffer) were used to monitor for contamination, and a positive PCR control (*L*. *donovani*, 100 pg/reaction) was included twice to check the PCR performance. All PCR runs were valid meaning that all positive controls were positive with Ct-values in the expected range and that all negative controls were negative (no Ct-values detected within 50 cycles). Ct-values are an indirect measure for the parasite load, with low Ct-values indicating high parasite loads, and high Ct-values indicating low parasite loads.

In case that a high Ct-value (> 38) was detected in a clinical sample for a single real-time PCR test, the sample was retested to confirm the positive result.

In case the clinical sample was negative for all real-time PCRs, a HBB PCR (targeting the human beta-globin gene) was done to detect human DNA to control for PCR inhibition, insufficient material or inefficient extraction as described before [32].

### Statistical analysis

The PCR data were entered into an Excel spreadsheet and analysed with R software (version 3.5.2, "Eggshell Igloo"- R Core Team) [33]. A CL suspected patient was identified as a true positive case if at least two of the six diagnostic tests (microscopy, ITS PCR, SSU PCR, Mary kDNA PCR, LC kDNA PCR and SL RNA PCR) were positive, which was used as the composite reference test, similarly to as described before [16]. Confidence intervals (CIs) for sensitivity and specificity were constructed using the Clopper-Pearson formula. The association between parasitic load and Ct-value was tested with ordinal ANOVA, using the ordPens package and illustrated with smoothers constructed with the mgcv package [34]. A type-I error (α) of 5% and equivalent 95% coverage for CIs was used for all analyses. Correlation between Ct-values of different methods was calculated using Pearson’s correlation coefficient and expressed as its associated R^2^ (which is the squared correlation, the percentage of variance explained or in common).

## Results

### Pre-analysis validation

The LC kDNA PCR validation showed reactivity for all Old World *Leishmania* species, except *L*. *major*. No New World *Leishmania* species were detected and no cross-reactivity was observed for *T*. *b*. *gambiense*, *T*. *b*. *rhodesiense*, *Mycobacterium leprae*, *M*. *lepromatosis* and *P*. *falciparum*. The analytical sensitivity of the assay was assessed to be at least 1 fg/reaction. Endemic and healthy non-endemic controls were all negative (*e*.*g*. no Ct-value detected up to 50 cycles).

The species was identified as *L*. *aethiopica* based on ITS-1 sequences found in the selected samples from 6 different area’s. Therefore, *Leishmania* will be here further referred to as *L*. *aethiopica*.

### Comparison of different CL diagnostic tests on SS samples

First, the positivity rate of SS samples was determined for each of the six diagnostic tests individually (microscopy and ITS, SSU, Mary kDNA, LC kDNA and SL RNA PCRs) (Table 1). Microscopy identified 46.8% of the subjects as *Leishmania* positive, after re-examination by a third reader due to 19.9% inter-observer discordant results. The molecular methods showed higher positivity rates, except the ITS PCR (19.8%). The SSU PCR resulted in 57.7% positive subjects while the Mary and LC kDNA PCRs showed a higher positivity rate of 89.2% and 90.1% respectively. The SL RNA PCR identified 85.6% positive SS RNA extracts.

**Table 1:** Overview of index tests with the number of positive and negative samples, and sensitivity and specificity compared to the composite reference (any two tests positive).

Test	Positive n (%)	Negative n (%)	Sensitivity % (95% CI)	Specificity % (95% CI)
Microscopy	52 (46.8)	59 (53.2)	52 (42–62)	100.0 (72–100)
ITS PCR	22 (19.8)	89 (80.2)	22 (14–31)	100.0 (72–100)
SSU PCR	64 (57.7)	47 (42.3)	64 (54–73)	100.0 (72–100)
Mary kDNA PCR	99 (89.2)	12 (10.8)	99 (95–100)	100.0 (72–100)
LC kDNA PCR	100 (90.1)	11 (9.9)	100 (96–100)	100.0 (72–100)
SL RNA PCR	95 (85.6)	16 (14.4)	94 (87–98)	91 (59–100)
Composite reference	100 (90.1)	11 (9.9)	NA	NA

Due to the lack of a gold standard reference test, the sensitivity and specificity of each method were calculated with a composite reference (Table 1), with a sample defined as truly positive if positive by at least two of the six index tests. This resulted in sensitivity for microscopy of 52% (95% CI, 42%-62%). The ITS PCR had the lowest sensitivity (22%; 95% CI, 14%-31%). The SSU, Mary and LC kDNA PCRs had a sensitivity of 64% (95% CI, 54–73%), 99% (95% CI, 95%-100%) and 100% (95% CI, 96%-100%) respectively. The SL RNA PCR displayed a sensitivity of 94% (95% CI, 87%-98%). Keeping in mind the limitation of having only a small set of samples that were negative with the composite reference test (n = 11), the specificity of all index tests was 100% except for the SL RNA PCR (91%) because the latter test identified one sample as positive at low Ct-value, which was not confirmed by the other assays.

When comparing overall, 10 out of the 111 SS samples were negative with all diagnostic methods, with one sample that was positive with only one test (the SL RNA PCR). In five of these negative samples, no human DNA was detected by the HBB PCR, indicating that PCR inhibition, insufficient sample start material or inefficient extraction cannot be excluded in these samples. All other 100 samples were confirmed as positive with at least one additional test (Table 2). Of these, four samples were positive by two tests, 20 by three tests, 29 by four tests, 35 by five tests and 12 were positive by all six tests.

**Table 2:** An overview of a number of tests and the number of skin scraping samples that gave a positive result for all observed combinations of index tests.

Nr of tests positive	Number of SS samples	Microscopy	ITS	SSU	Mary kDNA	LC kDNA	SL RNA
**0**	**10**						
**1**	**1**						**+**
**2**	**3**				**+**	**+**	
**2**	**1**	**+**				**+**	
**3**	**18**				**+**	**+**	**+**
**3**	**2**	**+**			**+**	**+**	
**4**	**17**			**+**	**+**	**+**	**+**
**4**	**10**	**+**			**+**	**+**	**+**
**4**	**2**		**+**		**+**	**+**	**+**
**5**	**27**	**+**		**+**	**+**	**+**	**+**
**5**	**8**		**+**	**+**	**+**	**+**	**+**
**6**	**12**	**+**	**+**	**+**	**+**	**+**	**+**

The two kDNA PCRs showed the highest agreement among each other with the same results except for one sample. The samples that were positive with the less sensitive tests (see Table 1), were all confirmed by tests with higher sensitivity, except one sample that was microscopically positive which was confirmed by the LC kDNA PCR only. This cumulative trend of samples being positive in more than one diagnostic test can also be seen.

**Fig 1:**
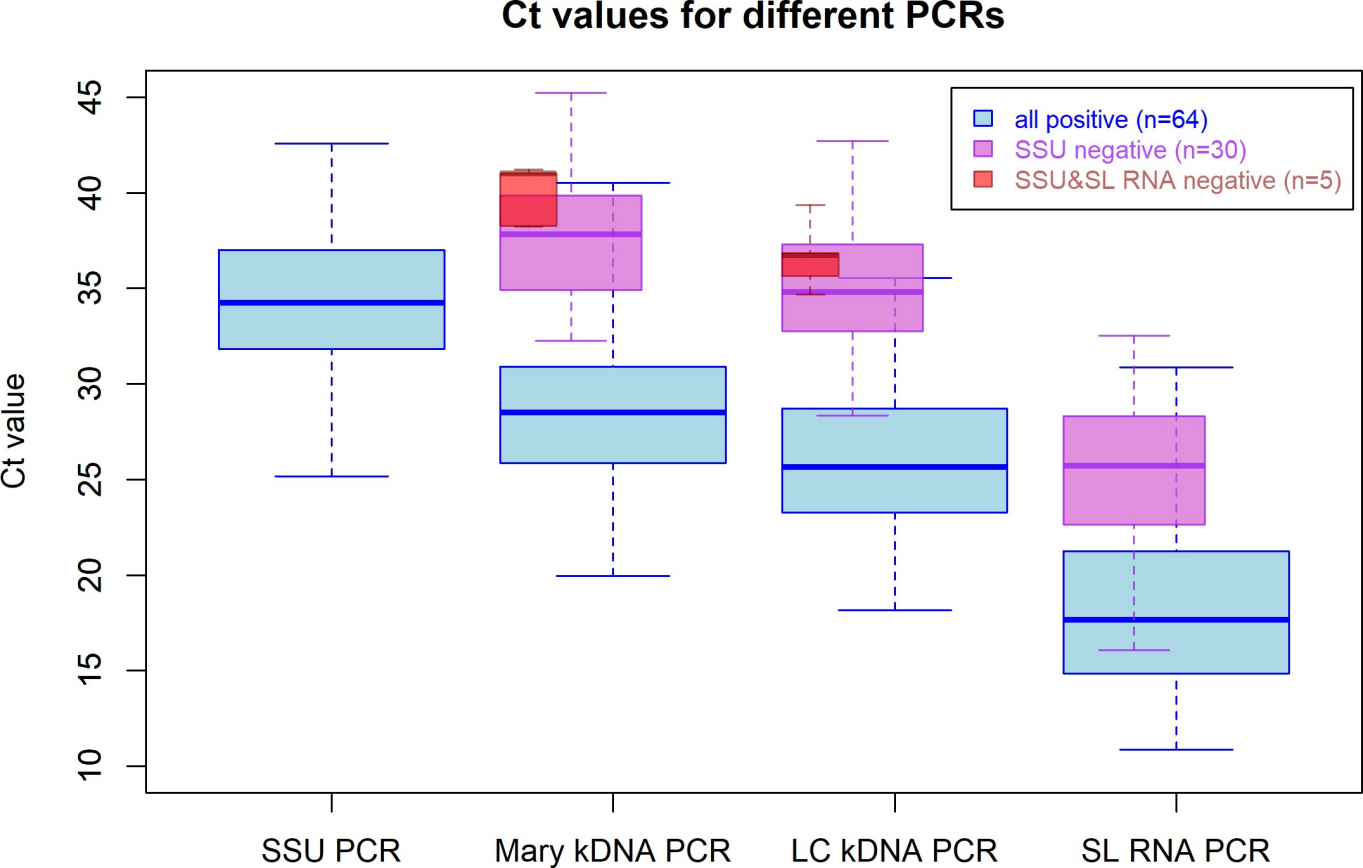
Range and median Ct-values for samples positive with the real-time PCR assays. Blue box plots represent samples that are positive in all four methods (n = 64); pink box plots present samples that were positive in the two kDNA PCRs and the SL RNA PCR, but negative in the SSU PCR (n = 30) and red box plots represent samples that were only positive with both kDNA PCRs (n = 5). The thick horizontal line in the box represents the median; the bottom and the top line of the box is the 25th and 75th percentile respectively.

The range of Ct-values for SS samples positive in all four real-time PCRs (n = 64, blue boxplots) are shown in Fig 1. Among these samples, the SSU PCR showed the highest median Ct-value (34.2), whereas Mary and LC kDNA PCRs had lower median Ct-values of 28.5 and 25.6 respectively. The SL RNA PCR assay had the lowest median Ct-value (17.7). Samples that were negative for the SSU PCR but positive with kDNA PCRs and SL RNA PCR (n = 30; pink boxplot), showed higher median Ct-values in the Mary kDNA (37.8), LC kDNA (34.8) and SL RNA (25.7) PCR assays than their counterparts that were also positive with SSU PCR. The five samples only positive by the two kDNA PCRs (red boxplots), gave median Ct-values of 41.0 for the Mary kDNA PCR and 36.7 for the LC kDNA PCR.

**Fig 2:**
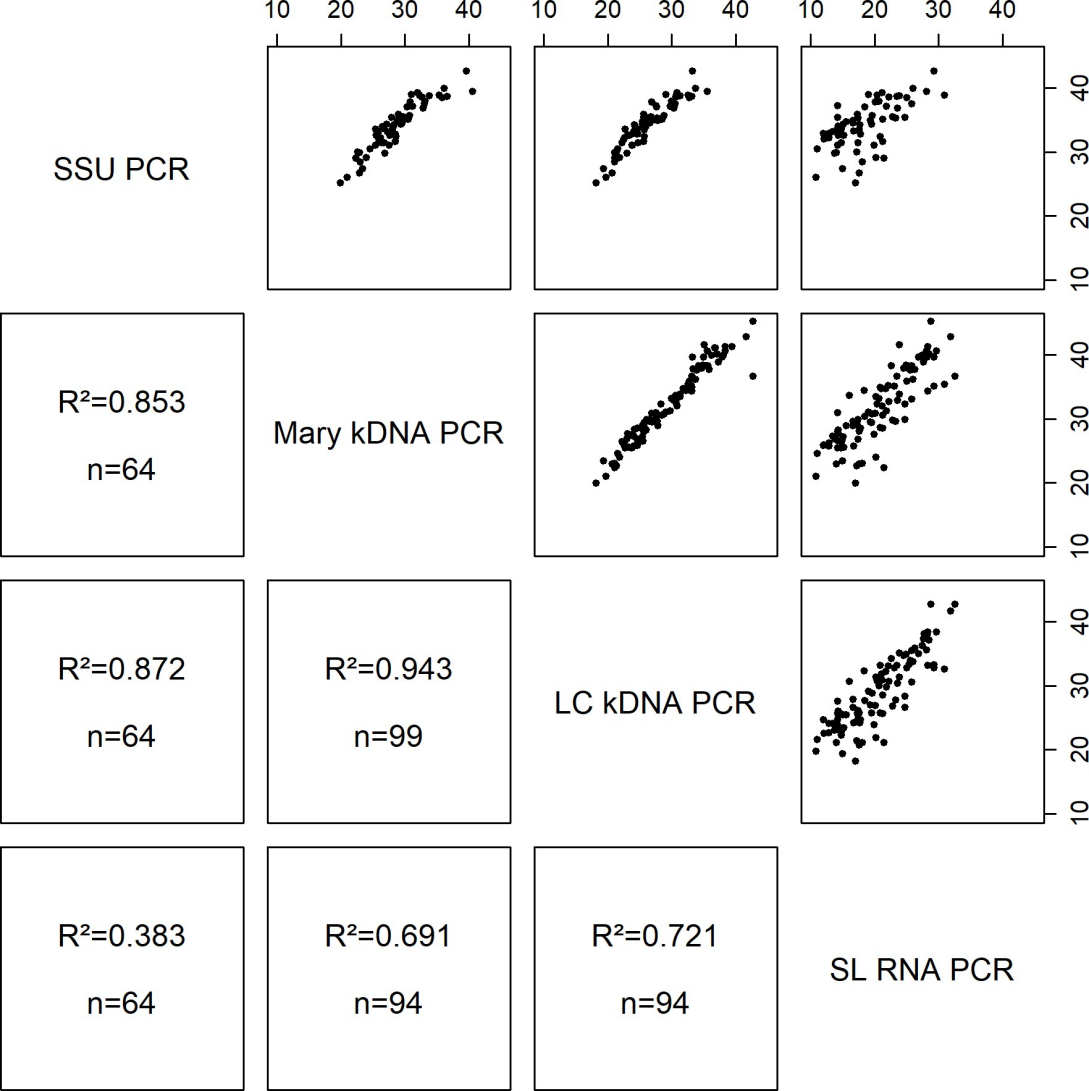
Comparison of Ct-values for the different real-time PCR assays on positive skin scraping DNA and RNA extracts. R^2^: squared correlation; n: number of samples positive in both PCR methods that were compared. The Ct-values are displayed on the X- and Y-axis.

Fig 2 shows the R^2^ correlation between Ct-values detected on SS DNA and RNA extracts with the different real-time PCR methods. The Mary kDNA and LC kDNA had the strongest relationship (R^2^ = 0.943, n = 99). The SSU PCR Ct-values were slightly less correlated with the ones from the LC kDNA (R^2^ = 0.872, n = 64) and Mary kDNA (R^2^ = 0.853, n = 64) assays. The correlation of the SL RNA PCR with the various DNA PCRs was lower: LC kDNA (R^2^ = 0.721, n = 94), Mary kDNA (R^2^ = 0.691, n = 94) and SSU PCR (R^2^ = 0.383, n = 64).

**Fig 3:**
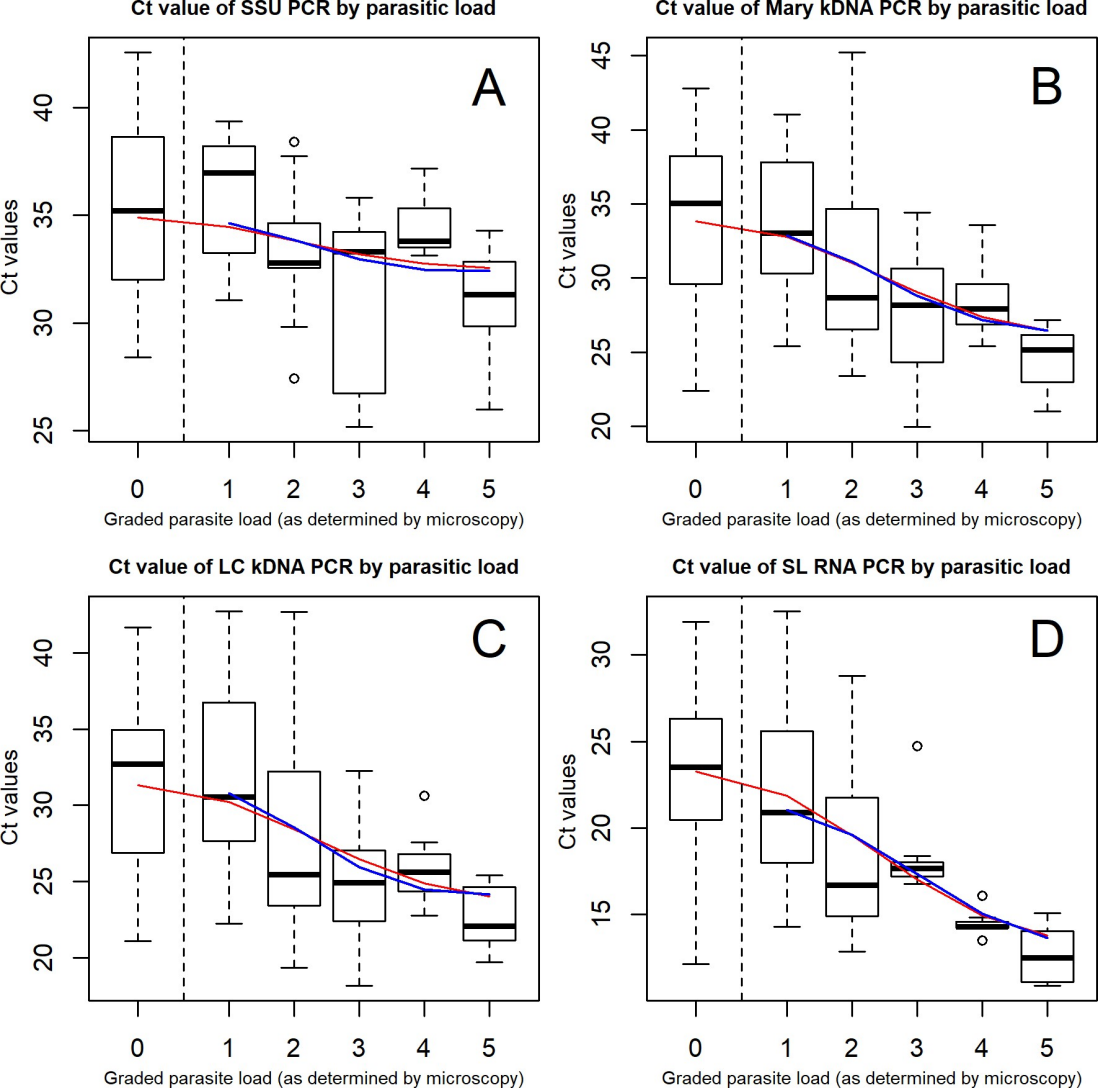
Boxplot for comparison of Ct-values with microscopy parasite load. The thick horizontal lines in the box represent the median; the bottom and top line of the box are the 25th and 75th percentile respectively. The red fitted line shows the trend of Ct-values of samples which were identified as positive by PCRs, including those negative by microscopy. The blue line shows the trend of Ct-values of the PCR by parasite load as of +1 in microscopy.

Ct-values of the real-time PCR assays were also compared with the parasitic load determined by microscopy (Fig 3). Overall, a clear trend was observed between the parasite load and the median Ct-values. The higher the parasite load, the lower the Ct-values and samples that were microscopy negative had the highest Ct-values in all PCRs. Statistical analysis showed that Ct-values were significantly associated with the parasite load for the SSU PCR (Fig 3A, p-value = 0.0253), and were more significant for the kDNA PCRs (Fig 3B, p-value: 0.0003; 3C p-value: 0.0004) and SL RNA PCR (Fig 3D, p-value: 0.0001).

### Comparison of SS and DBS sample types

**Fig 4:**
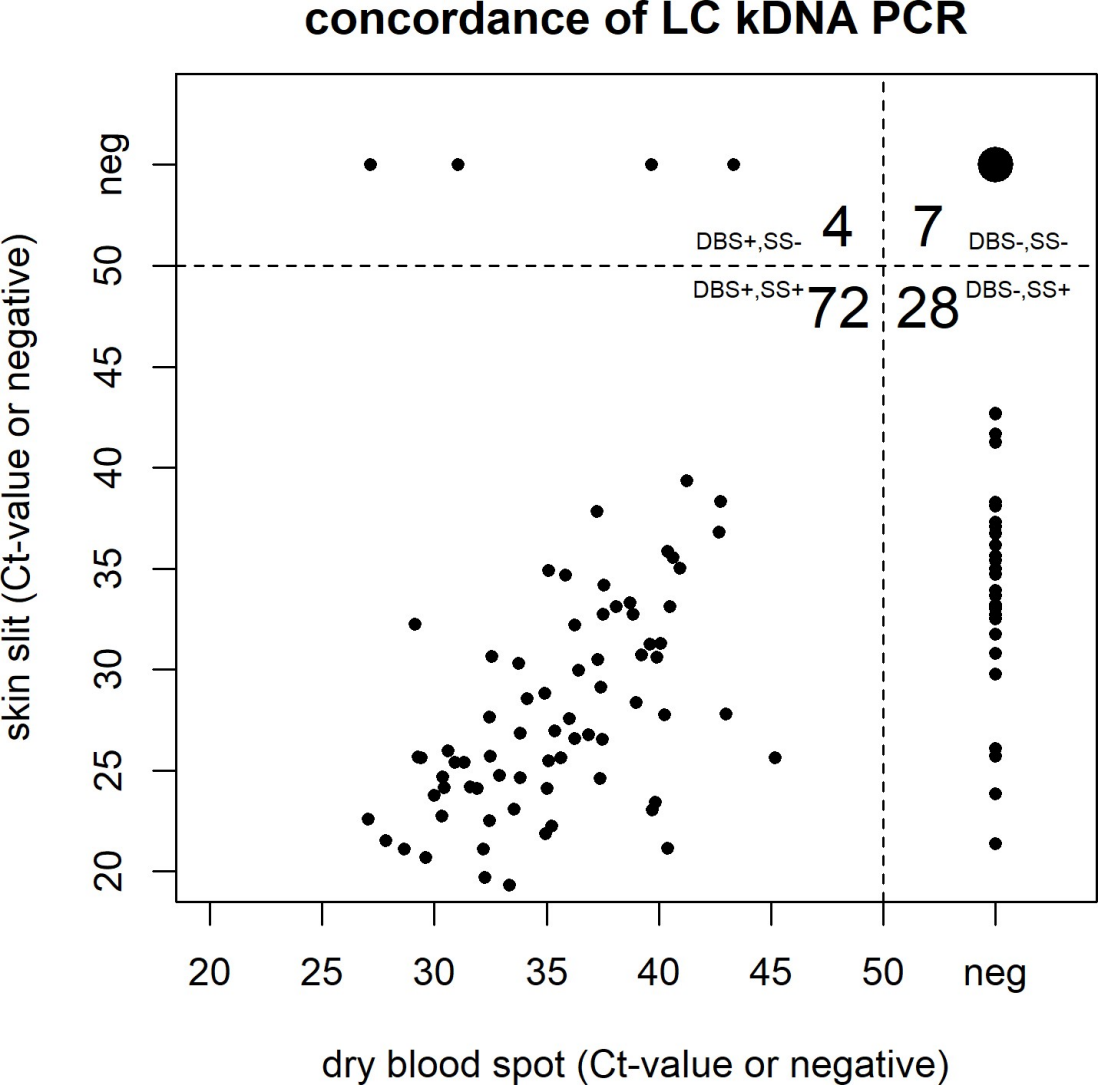
Comparison of Ct-values and concordance of Dry Blood Spots (DBS) versus Skin Scraping (SS) for the LC kDNA PCR.

To compare the two sampling methods, the PCRs with the highest sensitivity were also evaluated on DBS samples. The comparison of DBS and SS samples tested by the LC kDNA PCR is displayed in Fig 4 and those by the Mary kDNA PCR and SL RNA PCR are shown in S1 Fig. The DBS, collected from the same incision of the lesion, generally showed a lower positivity rate (76/111; 68.4%) compared to the SS. In particular, 28 subjects that were identified as positive by the LC kDNA PCR on SS (with Ct-values ranging between 21.4 and 42.6) were negative based on the DBS sample. Seven individuals were identified as negative by both sampling methods and 72 (65%) subjects were identified as positive in both sample types with Ct-values that were generally higher on DBS (ranging between 18.2 and 39.2 on SS and 27.1 and 42.0 on DBS). On the contrary, four subjects that were negative on SS were additionally identified as CL case based on the DBS sample with the LC kDNA PCR with Ct-values between 27.1 and 43.3. Of these four DBS samples, three were positive with the Mary kDNA PCR and two with the SL RNA PCR as well. Additionally, one more case was only detected on the DBS sample and not on the SS sample by the Mary kDNA PCR and SL RNA PCR (S1 Fig).

## Discussion

For neglected tropical diseases such as leishmaniasis, laboratory confirmation of clinical suspicion is mostly done by traditional methods (microscopy). The lack of sensitivity of this approach can hamper diagnosis and treatment. We therefore investigated the added value of PCR to accelerate its implementation in routine practice. We compared microscopy with five PCR assays including conventional and real-time formats with different gene targets on DNA or RNA extracts of two sample types from patients suspected of CL from a wide geographical area in the South of Ethiopia. The two sample types (SS and DBS) were collected from the same lesion and the DNA and RNA were simultaneously extracted from the same starting material. This approach avoids inter-lesional differences and reduces deviations due to different extraction methods which make the comparison as fair as possible.

Six samples from different zones were identified up to species level and were all *L*. *aethiopica*. However, this does not rule out that there were no *L*. *tropica* or *L*. *major* cases among the samples. As there are several reports of other *Leishmania* species isolated from sand flies and rodents in the country [35, 36], more large-scale molecular studies in different parts of Ethiopia are required to determine which species are causing CL and MCL.

Direct identification of amastigotes by microscopy on Giemsa-stained skin scraping smears is still the standard method for the diagnosis of CL in Ethiopia. Especially in endemic regions, it is widely available and the first-choice method [37] being familiar to lab staff and not expensive. In this study, the positivity rate of microscopy was 46.8% (Table 1) and lies within the range of 40% to 75% seen in other Old World CL endemic countries. The high inter-observer disagreement observed in this study demonstrates again that microscopy can be technically challenging [38–42] and requires the presence of a relatively high number of intact parasites [43]. This can be problematic in chronic lesions when patients present late [44] or with complex mucocutaneous CL [38], as parasite loads in these lesions are generally low [45]. Hence, PCR is reported to be superior to other methods for chronic lesions [46].

In contrast to microscopy, it is well-known that molecular tools can provide rapid, sensitive, accurate detection, quantification, and species identification depending on the target and design used [47–49]. In this study, we, therefore, compared the performance of well-known (SSU, kDNA) and less common (SL) PCR targets for CL detection. Since microscopy could not be used as the reference method, we applied a composite reference, similar to as described before [16, 50] to judge on the sensitivity of the different methods. Specificity was presented for completeness, but this result should be interpreted with care due to the low number of negative samples.

Of the five molecular assays in this study, the sensitivity of the conventional ITS PCR was lowest and unexpected, even lower than microscopy (Table 1). Literature showed various sensitivities of ITS for CL diagnosis ranging from 69.2% up to 96.6% [10, 20, 51, 52]. This poor performance might be explained by the use of 1/10 diluted DNA, the lower PCR reaction volume or the possibility of the parasite load in the samples of our study being at the limit of detection of the ITS assay. Moreover, the copy number of the ITS gene (20–200 copies) is much lower compared to the kDNA and SL RNA targets [10, 53] as demonstrated before [20, 52, 54]. However, when the ITS PCR does not provide the desirable sensitivity, a subsequent nested PCR could be performed to increase its performance [55]. Based on our experience, we would advise against the use of conventional or nested PCR formats for routine diagnostics, due to the higher workload and risk of post-PCR contamination. On the other hand, the amplicons of conventional PCRs are generally longer, and the universal ITS PCR therefore allows species identification by RFLP, sequencing or high-resolution melt technology [53, 56].

The SSU PCR targets a 115 bp-long highly conserved region [21] allowing broad use in *Leishmania* detection at the genus level, but without the ability of species discrimination. Although the SSU gene has similar copy numbers (20–400), the SSU PCR performed better than the ITS PCR as demonstrated here and before [57], probably due to its real-time format and shorter amplicon length. The PCR can amplify up to one single parasite in human blood [30], which relates to clinical disease [58]. However, in our study, the SSU PCR had a lower sensitivity than the kDNA PCRs as has been reported before [19, 20].

In this study, both kDNA PCRs identified the same subjects as CL positive, except one (Table 2). Studies done in Old World CL countries demonstrated high sensitivities for kDNA targeting PCRs ranging between 91.7% up to 100% [10, 20, 50, 59]. The kDNA minicircle sequence is by far the most often used target in studies on visceral [26, 32, 58] or New World leishmaniasis [19, 60]. With over 10,000 copies of minicircles per parasite, this PCR is more sensitive than the SSU [57] and ITS PCRs [10]. The Mary kDNA PCR was designed for species of the *L*. *donovani* complex group and some of the CL causing *Leishmania* species are not well detected [26]. We, therefore, designed a new PCR, the LC kDNA PCR, to improve amplification of *L*. *aethiopica* based on primers described first by Nuzum *et al*., [13] for symptomatic VL and used before by Nicolas and colleagues [25] for Old and New World CL species in mice and for differentiation of Old World CL species by melt curve analysis [61]. With its new probe-based format, the LC kDNA PCR showed the highest sensitivity among all PCRs, with lower Ct-values than the Mary kDNA PCR for *L*. *aethiopica* detection in CL suspected cases (Fig 1), illustrating its potential in Old World CL diagnosis. The potential of the kDNA PCR as a quantitative tool for treatment follow-up for (M) CL patients [62] is also of interest in clinical practice.

The skin slit RNA extracts were subjected to the pan-*Leishmania* SL RNA PCR [27] which performed well for CL diagnosis despite sample storage at -20°C without RNA stabilizing reagents and the use 1/10 diluted RNA. The assay performed better than the SSU and ITS PCRs, probably due to the high copy number and very short amplicon (39 bp). Only six out of 99 SS samples that were positive by both kDNA PCRs were not detected (Table 2). Overall, Ct-values were low compared to the other PCR assays as described earlier (Fig 1) [27]. Although more stringent storage conditions are generally needed for RNA [63, 64], it did not compromise assay performance in this study of field collected samples. RNA detection is also considered as a marker for viable parasites [65] although it has been demonstrated that longer targets are more indicative of viability than shorter amplicons [55]. The intercalator dye-based format of the SL RNA PCR assay is substantially cheaper than probe-based assays and thus beneficial for use in high throughput testing and epidemiological research.

One of the added values of PCR methods, in general, is that they can be applied to different types of clinical specimens [66–71]. The standard sample for CL diagnosis is a punch biopsy or skin scraping but less invasive sample collection methods have been studied [50, 71]. Lesion aspirates showed lower sensitivities compared to biopsies [47, 72] while filter paper lesion impression has a high sensitivity for ulcerative lesions and looks promising in New World CL [16, 50]. Collection of samples with filter paper is relevant for use in field conditions and to simplify transport.

In this study, a total of 102 of the 111 suspected cases were confirmed by PCR, 100 on SS and two additional ones on DBS. CL could not be diagnosed in only eight patients that were negative by all PCRs on both sample types, of which only a part could be explained by PCR inhibition, insufficient starting material or inefficient nucleic acid extraction [72]. We also found that the DBS performed less than SS and had a 28% lower positivity rate with the LC kDNA PCR and that the filter paper storage conditions were not ideal for RNA stability as demonstrated by the 38% lower positivity rate with the SL RNA PCR. Of note, the performance of PCR on DBS was still better than microscopy. It would be interesting to evaluate other sample collection methods like lesion impressions on filter paper or a tape stripping sampling method in future studies, which are also easy to perform in the field [73–75].

Overall, all four real-time PCR formats performed better than microscopy and the conventional ITS PCR, and Ct-values correlated well with the parasite load, making them valid for monitoring parasite quantities during follow-up [73]. The new LC kDNA PCR proved to be an excellent assay for CL diagnosis in Ethiopia. The lower-cost SL RNA detection represents a complementary tool which can be useful for high throughput studies. SS samples performed much better than DBS, but regarding sensitivity, PCR on DBS is still preferred above microscopy.

In Ethiopia, there is currently no comprehensive diagnostic algorithm that includes molecular methods and it would require additional infrastructure and training at centers nearby CL endemic sites. Therefore, on the basis of our results, we propose that at the primary health care level, microscopy can still be the first diagnostic method followed by treatment when positive. In case that microscopy is negative, the SS sample can be sent to the referral health facility where a kDNA PCR method is available. With this study, we therefore advocate for the implementation of PCR in routine care for CL diagnosis, and at least at the referral hospital level.

## Supporting information

S1 Fig Comparison of Ct values of DBS vs SS concordance for Mary kDNA PCR and SL RNA PCR. These scatterplots show the Ct-values of DBS vs. SS samples screened by the Mary kDNA and SL RNA PCRs. On top and on the right side of the graph, the Ct-values are shown for patients for whom only 1 out of the two tests was positive (indicated by “neg” on their respective axes). The number of patients in each of the pos/pos, pos/neg, neg/pos, neg/neg combos are shown in the upper right corner.
(TIF)

## References

[1] Alvar J, Vélez ID, Bern C, Herrero M, Desjeux P, Cano J, et al Leishmaniasis worldwide and global estimates of its incidence. PLoS One. 2012; 7(5):e35671 10.1371/journal.pone.0035671 22693548 PMC3365071

[2] Bray RS, Ashford RW, Bray MA. The parasite causing cutaneous leishmaniasis in Ethiopia. Trans R Soc Trop Med Hyg 1973;67(3):345–8. 10.1016/0035-9203(73)90111-9 4778189

[3] Assefa A. Leishmaniasis in Ethiopia: A systematic review and meta-analysis of prevalence in animals and humans. Heliyon. 2018;4(8):e00723 10.1016/j.heliyon.2018.e00723 30101202 PMC6082994

[4] Federal Democratic Republic of Ethiopia. National Neglected Tropical Diseases Master Plan, Second Edition 2016–2020. 5 2016 Federal Ministry of Health, Ethiopia 2016.

[5] Yanik M, Gurel MS, Simsek Z, Kati M. The psychological impact of cutaneous leishmaniasis. Clin Exp Dermatol. 2004; 29(5):464–7. 10.1111/j.1365-2230.2004.01605.x 15347324

[6] Masmoudi A, Hariz W, Marrekchi S, Amouri M, Turki H. Old World cutaneous leishmaniasis: Diagnosis and treatment. J Dermatol Case Rep. 2013;7(2):31–41. 10.3315/jdcr.2013.1135 23858338 PMC3710675

[7] Fikre H, Mohammed R, Atinafu S, van Griensven J, Diro E. Clinical features and treatment response of cutaneous leishmaniasis in North-West Ethiopia. Trop Med Int Heal. 2017;22(10):1293–301. 10.1111/tmi.12928 28712122

[8] van Henten S, Adriaensen W, Fikre H, Akuffo H, Diro E, Hailu A, et al Cutaneous Leishmaniasis Due to *Leishmania aethiopica*. EClinicalMedicine. 2018;6:69–81. 10.1016/j.eclinm.2018.12.009 31193672 PMC6537575

[9] Goto H, Lindoso JAL. Current diagnosis and treatment of cutaneous and mucocutaneous leishmaniasis. Expert Rev Anti Infect Ther. 2010;8(4):419–33. 10.1586/eri.10.19 20377337

[10] Bensoussan E, Nasereddin A, Jonas F, Schnur LF, Jaffe CL. Comparison of PCR assays for diagnosis of cutaneous leishmaniasis. J Clin Microbiol. 2006;44(4):1435–9. 10.1128/JCM.44.4.1435-1439.2006 16597873 PMC1448629

[11] Kebede A, Beyene D, Yenew B, Diriba G, Mehamd Z, Alemu A, et al Monitoring quality indicators for the Xpert MTB/RIF molecular assay in Ethiopia. PLoS One. 2019;14(11): e0225205 10.1371/journal.pone.0225205 31714934 PMC6850546

[12] El-Sadr WM, Rabkin M, Nkengasong J, Birx DL. Realizing the potential of routine viral load testing in sub-Saharan Africa: J Int AIDS Soc. 2017;20(7):e25010 10.1002/jia2.25010 29130621 PMC5978658

[13] Nuzum E, White F, Thakur C, Dietze R, Wages J, Grogl M, et al Diagnosis of symptomatic visceral leishmaniasis by use of the polymerase chain reaction on patient blood. J Infect Dis. 1995;171(3):751–4. 10.1093/infdis/171.3.751 7876635

[14] Galluzzi L, Ceccarelli M, Diotallevi A, Menotta M, Magnani M. Real-time PCR applications for diagnosis of leishmaniasis. Parasit Vectors. 2018;11(1):273 10.1186/s13071-018-2859-8 29716641 PMC5930967

[15] Marques MJ, Volpini AC, Genaro O, Mayrink W, Romanha AJ. Simple form of clinical sample preservation and leishmania DNA extraction from human lesions for diagnosis of American cutaneous leishmaniasis via polymerase chain reaction. Am J Trop Med Hyg. 2001;65(6):902–6. 10.4269/ajtmh.2001.65.902 11791996

[16] Boggild AK, Valencia BM, Espinosa D, Veland N, Ramos AP, Arevalo J, et al Detection and Species Identification of *Leishmania* DNA from Filter Paper Lesion Impressions for Patients with American Cutaneous Leishmaniasis. Clin Infect Dis. 2010; 50(1):e1–6. 10.1086/648730 19947858

[17] Romero GAS, Noronha EF, Pirmez C, do Espirito Santo Silva Pires F, Fernandes O, Nehme NS, et al Sensitivity and reproducibility of a PCR assay for *Leishmania* detection using skin biopsy imprints on filter paper. Acta Trop. 2009;109(1):74–7. 10.1016/j.actatropica.2008.10.003 18996076

[18] Kitchen M, Marti H, Schmuth M. Cutaneous leishmaniasis: PCR of filter paper blots from an ulcer base is an alternative to biopsy. J Dtsch Dermatol Ges. 2018;16(6):772–4. 10.1111/ddg.13537 29762895

[19] Conter CC, Mota CA, dos Santos BA, de Souza Braga L, de Souza Terron M, Navasconi TR, et al PCR primers designed for new world *Leishmania*: A systematic review. Exp Parasitol. 2019;207:107773 10.1016/j.exppara.2019.107773 31605671

[20] Koltas IS, Eroglu F, Uzun S, Alabaz D. A comparative analysis of different molecular targets using PCR for diagnosis of old world leishmaniasis. Exp Parasitol. 2016;164:43–8. 10.1016/j.exppara.2016.02.007 26896641

[21] van Eys Schoone GJ, Kroon NCM Ebeling SB. Sequence analysis of small subunit ribosomal RNA genes and its use for detection and identification of *Leishmania* parasites. Mol Biochem Parasitol. 1992;51(1):133–42. 10.1016/0166-6851(92)90208-2 1565128

[22] El Tai NO, Osman OF, El Fari M, Presber W, Scho¨nian G. Genetic heterogeneity of ribosomal internal transcribed spacer (ITS) in clinical samples of *Leishmania donovani* spotted on filter paper as revealed by single-strand conformation polymorphisms (SSCP) and sequencing. Trans R Soc Trop Med Hyg. 2000; 94:575–9. 10.1016/s0035-9203(00)90093-2 11132393

[23] Hassan Q., Ghosh A., Ghosh S., Gupta M., Basu D., Mallik K., & Adhya S. Enzymatic amplification of mini-exon-derived RNA gene spacers of *Leishmania donovani*: Primers and probes for DNA diagnosis. Parasitology. 1993;107(5):509–517. 10.1017/s0031182000068086 8295790

[24] Marfurt J, Nasereddin A, Niederwieser I, Jaffe CL, Beck H, Felger I. Identification and Differentiation of *Leishmania* Species in Clinical Samples by PCR Amplification of the Miniexon Sequence and Subsequent Restriction Fragment Length Polymorphism Analysis. J Clin Microbiol. 2003;41(7):3147–53. 10.1128/jcm.41.7.3147-3153.2003 12843055 PMC165364

[25] Nicolas L, Prina E, Lang T. Real-Time PCR for Detection and Quantitation of *Leishmania* in Mouse tissue. Journal of Clinical Microbiology. 2002;40(5):1666–9. 10.1128/jcm.40.5.1666-1669.2002 11980939 PMC130941

[26] Mary C, Faraut F, Lascombe L, Dumon H. Quantification of *Leishmania infantum* DNA by a real-time PCR assay with high sensitivity. J Clin Microbiol. 2004;42(11):5249–55. 10.1128/JCM.42.11.5249-5255.2004 15528722 PMC525214

[27] Eberhardt E, Van den Kerkhof M, Bulté D, Mabille D, Van Bockstal L, Monnerat S, et al Evaluation of a Pan-*Leishmania* Spliced-Leader RNA Detection Method in Human Blood and Experimentally Infected Syrian Golden Hamsters. J Mol Diagnostics. 2018; 20(2):253–63. 10.1016/j.jmoldx.2017.12.003 29355825

[28] World Health Organization. Basic Laboratory methods in medical parasitology first Edn. 1991. Geneva. 10.1016/0169-4758(92)90312-p

[29] World Health Organization. Control of the Leishmaniasis. Report of a meeting of the WHO Expert Committee on the control of leishmaniasis. 2010 World Health Organ Tech Rep Ser 949.

[30] Pareyn M, Van den Bosch E, Girma N, van Houtte N, Van Dongen S, Van der Auwera G, et al Ecology and seasonality of sandflies and potential reservoirs of cutaneous leishmaniasis in Ochollo, a hotspot in southern Ethiopia. PLoS Negl Trop Dis. 2019;13(8):e0007667 10.1371/journal.pntd.0007667 31425506 PMC6715250

[31] Deborggraeve S, Boelaert M, Rijal S, De Doncker S, Dujardin JC, Herdewijn P, et al Diagnostic accuracy of a new *Leishmania* PCR for clinical visceral leishmaniasis in Nepal and its role in diagnosis of disease. Trop Med Int Heal. 2008;13(11):1378–83. 10.1111/j.1365-3156.2008.02154.x 18803611

[32] Cnops L, Van Esbroeck M, Bottieau E, Jacobs J. Giemsa-stained thick blood films as a source of DNA for *Plasmodium* species-specific real-time PCR. Malar J. 2010;9:370 10.1186/1475-2875-9-370 21176207 PMC3016375

[33] R Core Team (2018). R: A language and environment for statistical computing. R Foundation for Statistical Computing, 2018; Vienna, Austria URL https://www.R-project.org/.

[34] Wood S.N. Generalized Additive Models: An Introduction with R (2nd edition). 2017 Chapman and Hall/CRC Press.

[35] Gebre-Michael, Balkew M., Ali A., Ludovisi A., Gramiccia M. The isolation of *Leishmania tropica* and *L*. *aethiopica* from *Phlebotomus* (*Paraphlebotomus*) species (Diptera: Psychodidae) in the Awash Valley, northeastern Ethiopia. Trans R Soc Trop Med Hyg. 2004;98(1):64–70. 10.1016/s0035-9203(03)00008-7 14702839

[36] Kassahun A, Sadlova J, Dvorak V, Kostalova T, Rohousova I, Frynta D, et al Detection of *Leishmania donovani* and *L*. *tropica* in Ethiopian wild rodents. Acta Trop. 2015;145:39–44. 10.1016/j.actatropica.2015.02.006 25700710

[37] Palma G., and Gutierrez Y. Laboratory Diagnosis of *Leishmania*. Clinics in Laboratory Medicine; 1991;11(4). 10.1016/s0272-2712(18)30527-4 1802528

[38] Mouttaki T, Morales-Yuste M, Merino-Espinosa G, Chiheb S, Fellah H, Martin-Sanchez J, et al Molecular diagnosis of cutaneous leishmaniasis and identification of the causative *Leishmania* species in Morocco by using three PCR-based assays. Parasit Vectors. 2014; 7(1). 10.1186/s13071-014-0467-9 25189460 PMC4161773

[39] Eroglu F, Uzun S, Koltas IS. Comparison of clinical samples and methods in chronic cutaneous leishmaniasis. Am J Trop Med Hyg. 2014;91(5):895–900. 10.4269/ajtmh.13-0582 25223940 PMC4228882

[40] Al-hucheimi SN, Sultan BA, Al-Dhalimi MA. A comparative study of the diagnosis of Old World cutaneous leishmaniasis in Iraq by polymerase chain reaction and microbiologic and histopathologic methods. Int J Dermatol. 2009;48(4):404–8. 10.1111/j.1365-4632.2009.03903.x 19335428

[41] Rasti S, Ghorbanzadeh B, Kheirandish F, Mousavi SG, Pirozmand A, Hooshyar H, et al Comparison of Molecular, Microscopic, and Culture Methods for Diagnosis of Cutaneous Leishmaniasis. J Clin Lab Anal. 2016;30(5):610–5. 10.1002/jcla.21910 26891976 PMC6807126

[42] Pourmohammadi B, Motazedian M, Hatam G, Kalantari M, Habibi P, Sarkari B. Comparison of three methods for diagnosis of cutaneous leishmaniasis. Iran J Parasitol. 2010;5(4):1–8. 22347259 PMC3279850

[43] Chargui N, Bastien P, Kallel K, Haouas N, Akrout FM, Masmoudi A, et al Usefulness of PCR in the diagnosis of cutaneous leishmaniasis in Tunisia. Trans R Soc Trop Med Hyg. 2005;99(10):762–8. 10.1016/j.trstmh.2005.06.002 16095641

[44] Laskay T, Mikó TL, Negesse Y, Solbach W, Röllinghoff M, Frommel D. Detection of cutaneous leishmania infection in paraffin-embedded skin biopsies using the polymerase chain reaction. Trans R Soc Trop Med Hyg. 1995;89(3):273–5. 10.1016/0035-9203(95)90537-5 7660431

[45] Arnold P R, Lord NP, Smith AN, Bybee SM. The Effects of Non-Ideal Temperature Regimes on RNA Quality from Samples Stored in RNAlater-like Buffer: An Attempt to Replicate Field Conditions. J Anal Mol Tech. 2016;2(1):1–8. 10.13188/2474-1914.1000006

[46] Weigle KA, Labrada LA, Lozano C, Santrich C, Barker DC. PCR-based diagnosis of acute and chronic cutaneous leishmaniasis caused by *Leishmania* (*Viannia*). J Clin Microbiol. 2002;40(2):601–6. 10.1128/jcm.40.2.601-606.2002 11825977 PMC153366

[47] Valones MAA, Guimarães RL, Brandão LAC, De Souza PRE, De Albuquerque Tavares Carvalho A, Crovela S. Principles and applications of polymerase chain reaction in medical diagnostic fields: A review. Brazilian J Microbiol. 2009;40(1):1–11. 10.1590/S1517-83822009000100001 24031310 PMC3768498

[48] Dwivedi S, Purohit P, Misra R, Pareek P, Goel A, Khattri S, et al Diseases and Molecular Diagnostics: A Step Closer to Precision Medicine. Indian J Clin Biochem. 2017;32(4):374–98. 10.1007/s12291-017-0688-8 29062170 PMC5634985

[49] Mesa LE, Manrique R, Muskus C, Robledo SM. Test accuracy of polymerase chain reaction methods against conventional diagnostic techniques for Cutaneous Leishmaniasis (CL) in patients with clinical or epidemiological suspicion of CL: Systematic review and meta-analysis. PLoS Negl Trop Dis. 2020;14(1):e0007981 10.1371/journal.pntd.0007981 31961871 PMC6994169

[50] Boggild AK, Ramos AP, Valencia BM, Veland N, Calderon F, Arevalo J, et al Diagnostic performance of filter paper lesion impression PCR for secondarily infected ulcers and nonulcerative lesions caused by cutaneous leishmaniasis. J Clin Microbiol. 2011;49(3):1097–100. 10.1128/JCM.02457-10 21177908 PMC3067679

[51] El-Beshbishy HA, Al-Ali KH, El-Badry AA. Molecular characterization of cutaneous leishmaniasis in Al-Madinah Al-Munawarah province, western Saudi Arabia. Int J Infect Dis. 2013;17(5):e334–8. 10.1016/j.ijid.2012.11.015 23253909

[52] Nasereddin A, Salant H, Abdeen Z. Feline leishmaniasis in Jerusalem: Serological investigation. Vet Parasitol. 2008;158(4):364–9. 10.1016/j.vetpar.2008.09.022 18986768

[53] Odiwuor S, De Doncker S, Maes I, Dujardin JC, Van der Auwera G. Natural *Leishmania donovani/Leishmania aethiopica* hybrids identified from Ethiopia. Infect Genet Evol 2011;11(8):2113–8. 10.1016/j.meegid.2011.04.026 21558020

[54] Albuquerque A, Campino L, Cardoso L. Evaluation of four molecular methods to detect Leishmania infection in dogs. 2017;1–5. 10.1186/s13071-017-2002-2 28285595 PMC5346836

[55] Schönian G, Nasereddin A, Dinse N, Schweynoch C, Schallig HDFH, Presber W, et al PCR diagnosis and characterization of *Leishmania* in local and imported clinical samples. Diagn Microbiol Infect Dis. 2003;47(1):349–58. 10.1016/s0732-8893(03)00093-2 12967749

[56] Talmi-Frank D, Nasereddin A, Schnur LF, Schönian G, Töz SO, Jaffe CL, Baneth G. Detection and identification of old world *Leishmania* by high resolution melt analysis. PLoS Negl Trop Dis. 2010;4(1):e581 10.1371/journal.pntd.0000581 20069036 PMC2797090

[57] De Paiva Cavalcanti M, Dantas-Torres F, Da Cunha Gonçalves de Albuquerque S, Silva de Morais RC, de Brito MEF, Otranto D, et al Quantitative real time PCR assays for the detection *of Leishmania (Viannia) braziliensis* in animals and humans. Mol Cell Probes. 2013;27(3–4):122–8. 10.1016/j.mcp.2013.01.003 23402826

[58] Mary C, Faraut F, Drogoul MP, Xeridat B, Schleinitz N, Cuisenier B, et al Reference values for *Leishmania infantum* parasitemia in different clinical presentations: Quantitative polymerase chain reaction for therapeutic monitoring and patient follow-up. Am J Trop Med Hyg. 2006;75(5):858–63. 10.4269/ajtmh.2006.75.858 17123977

[59] Hitakarun A, Tan-Ariya P, Siripattanapipong S, Mungthin M, Piyaraj P, Naaglor T, et al Comparison of PCR methods for detection of *Leishmania siamensis* infection. Parasit Vectors. 2014;7(1). 10.1186/s13071-014-0467-9 25274259 PMC4188918

[60] De Brujin MH, Barker DC. Diagnosis of New World leishmaniasis: specific detection of species of the *Leishmania braziliensis* complex by amplification of kinetoplast DNA. Acta Trop. 1992; 52(1):45–58. 10.1016/0001-706x(92)90006-j 1359760

[61] Nicolas L, Milon G, Prina E. Rapid differentiation of Old World *Leishmania* species by LightCycler polymerase chain reaction and melting curve analysis. J Microbiol Methods. 2002;51(3):295–9. 10.1016/s0167-7012(02)00099-4 12223289

[62] Jara M, Adaui V, Valencia BM, Martinez D, Alba M, Castrillon C, et al Real-time PCR assay for detection and quantification of *Leishmania* (*Viannia*) organisms in skin and mucosal lesions: Exploratory study of parasite load and clinical parameters. J Clin Microbiol. 2013;51(6):1826–33. 10.1128/JCM.00208-13 23554201 PMC3716068

[63] Eberhardt E, Hendrickx R, Van den Kerkhof M, Monnerat S, Alves F, Hendrickx S, et al Comparative evaluation of nucleic acid stabilizing reagents for RNA- and DNA-based *Leishmania* detection in blood as proxy for visceral burdens. J Microbiol Methods. 2020; 173:105935 10.1016/j.mimet.2020.105935 32376283

[64] Romero I, Téllez J, Suárez Y, Cardona M, Figueroa R, Zelazny A, et al Viability and burden of *Leishmania* in extra lesional sites during human dermal leishmaniasis. PLoS Negl Trop Dis. 2010;4(9). 10.1371/journal.pntd.0000819 20856851 PMC2939031

[65] Keer JT, Birch L. Molecular methods for the assessment of bacterial viability. J Microbiol Methods. 2003;53(2):175–83. 10.1016/s0167-7012(03)00025-3 12654489

[66] Al-Jawabreh A, Schoenian G, Hamarsheh O, Presber W. Clinical diagnosis of cutaneous leishmaniasis: A comparison study between standardized graded direct microscopy and ITS1-PCR of Giemsa-stained smears. Acta Trop. 2006;99(1):55–61. 10.1016/j.actatropica.2006.07.001 16920056

[67] Azmi SZ, Latif MT, Ismail AS, Juneng L, Jemain AA. Trend and status of air quality at three different monitoring stations in the Klang Valley, Malaysia. Air Qual Atmos Heal. 2010;3(1):53–64. 10.1007/s11869-009-0051-1 20376168 PMC2844963

[68] Lachaud L, Marchergui-Hammami S, Chabbert E, Dereure J, Dedet JP, Bastien P. Comparison of six PCR methods using peripheral blood for detection of canine visceral leishmaniasis. J Clin Microbiol. 2002;40(1):210–5. 10.1128/jcm.40.1.210-215.2002 11773118 PMC120090

[69] Rodriguez A., Chen P., Oliver H., Abrams J.M. Unrestrained caspase-dependent cell death caused by loss of *Diap1* function requires the *Drosophila* Apaf-1 homolog, *Dark*. EMBO J. 2002;21(9):2189–2197. 10.1093/emboj/21.9.2189 11980716 PMC125994

[70] Vega-Lopez F. Diagnosis of cutaneous leishmaniasis. Current Opinion in Infectious Diseases. 2003;16(2):97–101. 10.1097/00001432-200304000-00006 12734442

[71] Matsumoto T. Hashiguchi Y., Gomez E.A., Calvopina M.H., Nonaka S., Saya H., Mimori T.. Comparison of PCR results using scrape/exudate, syringe-sucked fluid and biopsy samples for diagnosis of cutaneous leishmaniasis in Ecuador, Trans Roy Soc Trop Med Hyg. 1999;93(6):606–7. 10.1016/s0035-9203(99)90065-2 10717745

[72] Cnops L, Boderie M, Gillet P, Van Esbroeck M, Jacobs J. Rapid diagnostic tests as a source of DNA for *Plasmodium* species-specific real-time PCR. Malar J. 2011;10:1–7. 10.1186/1475-2875-10-1 21435256 PMC3075219

[73] Fekri-Soofiabadi M, Fekri M, Moradabadi A, Vahidi R, Shamsi-Meymandi S, Dabiri D, et al Ability of real-Time PCR for differential diagnosis of various forms of cutaneous leishmaniasis: A comparative study with histopathology. BMC Res Notes. 2019;12(1):1–5. 10.1186/s13104-018-4038-6 31547842 PMC6757515

[74] Taslimi Y, Sadeghipour P, Habibzadeh S, Mashayekhi V, Lane ME, Kropf P, et al A novel non-invasive diagnostic sampling technique for cutaneous leishmaniasis. PLoS Negl Trop Dis. 2017;11(7):e0005750 10.1371/journal.pntd.0005750 28704463 PMC5526608

[75] Khan NH, Bari AU, Hashim R, Khan I, Muneer A, Shah A, et al Cutaneous Leishmaniasis in Khyber Pakhtunkhwa Province of Pakistan: Clinical Diversity and Species-Level Diagnosis. Am J Trop Med Hyg. 2016;95(5):1106–1114. 10.4269/ajtmh.16-0343 27601518 PMC5094225

