# Supplementary figures and images for: Evaluation of conventional and four real-time PCR methods for the detection of Leishmania on field-collected samples in Ethiopia
Source: PLoS Negl Trop Dis. 2021 Jan 12;15(1):e0008903. doi: 10.1371/journal.pntd.0008903 (PMC7802924; doi:10.1371/journal.pntd.0008903)

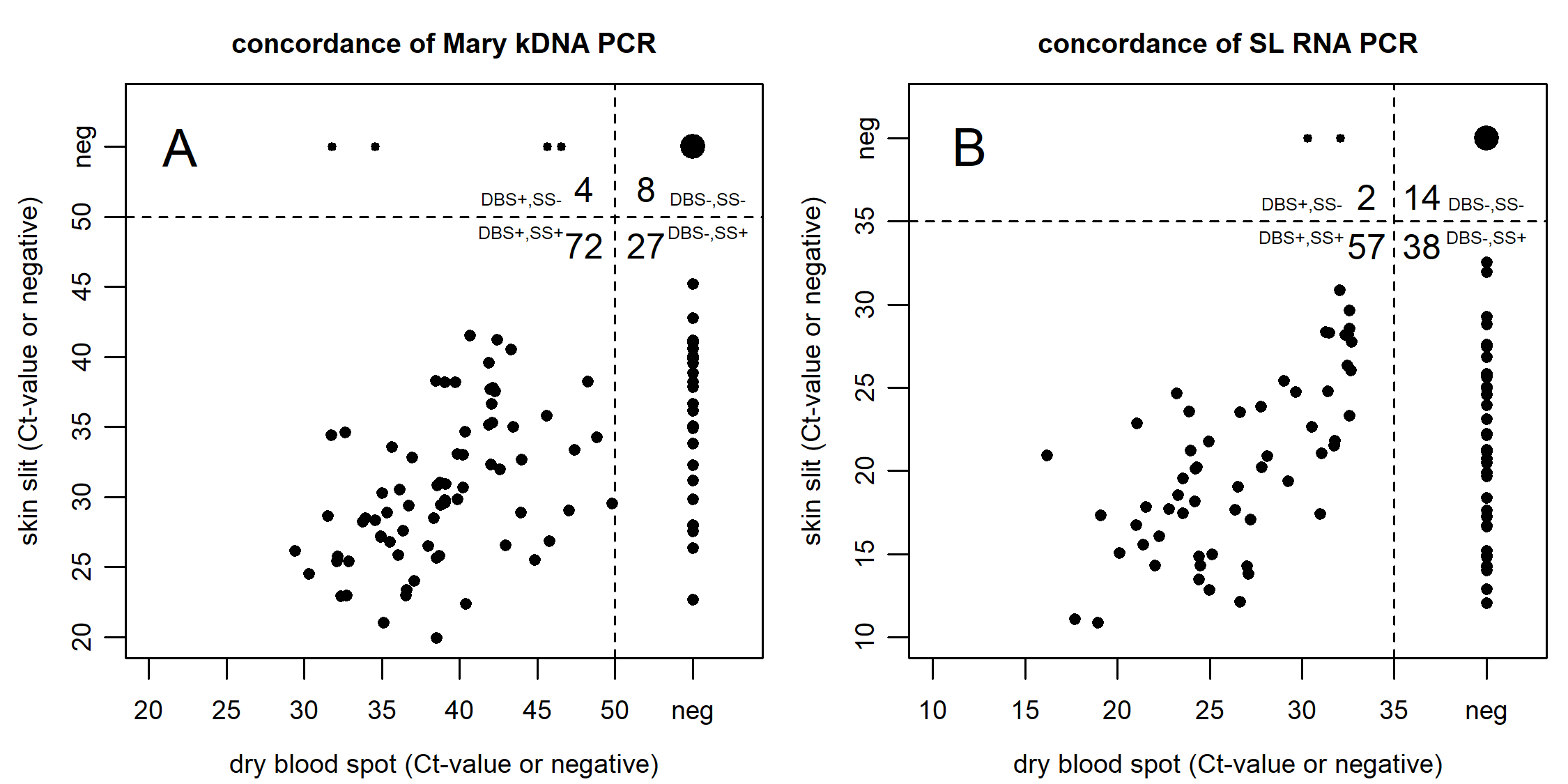

Supplement: S1 Fig — These scatterplots show the Ct-values of DBS vs. SS samples screened by the Mary kDNA and SL RNA PCRs. On top and on the right side of the graph, the Ct-values are shown for patients for whom only 1 out of the two tests was positive (indicated by “neg” on their respective axes). The number of patients in each of the pos/pos, pos/neg, neg/pos, neg/neg combos are shown in the upper right corner. (TIF) [file pntd.0008903.s001.tif]
